# The sol-gel entrapment of noble metals in hybrid silicas: a molecular insight

**DOI:** 10.1186/1752-153X-7-161

**Published:** 2013-09-30

**Authors:** Alexandra Fidalgo, Rosaria Ciriminna, Luís Lopes, Valerica Pandarus, François Béland, Laura M Ilharco, Mario Pagliaro

**Affiliations:** 1 Centro de Química-Física Molecular and IN - Institute of Nanoscience and Nanotechnology, Instituto Superior Técnico, Complexo I, Av. Rovisco Pais 1, Lisboa 1049-001, Portugal; 2 Istituto per lo Studio dei Materiali Nanostrutturati, CNR, via U. La Malfa 153, Palermo 90146, Italy; 3 SiliCycle Inc., 2500, Parc-Technologique Blvd, Quebec City, Quebec G1P 4S6, Canada

**Keywords:** Sol–gel, Encapsulation, Heterogeneous catalysis, Metal nanoparticle, ORMOSIL

## Abstract

**Background:**

Why are metal nanoparticles sol-gel entrapped in ORMOSIL so active and stable? In other words, why ORMOSIL-entrapped metal nanoparticles are more active and selective than many heterogenized counterparts, including silica-entrapped noble metals?

**Results:**

Unveiling specific interactions between MNPs and the molecular structure of ORMOSIL, this work investigates subtle structural aspects through DRIFT spectroscopy.

**Conclusions:**

The results point to interactions between entrapped Pd and Pt nanocrystallites with the organosilica sol-gel cages similar to those taking place in enzymes.

## Introduction

Supported metal nanoparticles (MNPs) catalyze a number of reactions of enormous relevance in the petrochemical industry such as hydrogenation, epoxidation and monomer synthesis [1]. In the last two decades, the use of supported metal nanoparticles in synthetic organic chemistry has been extensively investigated in light of their ability to catalyze a range of chemical reactions [2], including asymmetric syntheses [3]. The aim was, and is, to apply heterogeneous catalysis to the synthesis of pharmaceutical and fine chemical products, for which the amount of effluent per tonne of product is orders of magnitude higher than that for a commodity chemical [4]. As a results of these efforts a number of new solid catalysts and green chemical processes are slowly being adopted by industry [5]; despite many remarkable research achievements like, as representative example, Cu ions immobilized on functionalized silica as recyclable and truly heterogeneous catalyst for the homocoupling of terminal alkynes in the presence of oxygen only [6].

In general, the atomic structure of the exposed surfaces of the active “naked” nanoparticles is made of plentiful unsaturated sites capable to adsorb and catalyze conversion of the reactants [7]. For example, Pd nanoparticles, which are well known for their catalytic activities, can easily aggregate to form Pd-black because of the very high surface energy of palladium [8]. Many efforts have been devoted to develop sinter-proof catalysts using, for example, pre-prepared colloidal metal nanoparticles with tuned size, shape and composition that are then “embedded” by porous support shells [9].

In principle, the heterogenization of MNPs should prevent the tendency of atoms of “naked” MNPs to aggregate into a bulk material due to their high surface energies, which results in rapid decrease in their intrinsic catalytic activity and selectivity over time [10]. Unfortunately, however, most heterogeneous catalysts reported in the literature, and especially palladium-based catalysts [11], act as reservoir for MNPs that are leached in solution where they catalyze reaction, but also rapidly aggregate resulting in spent catalyst of poor residual activity.

Sintering is caused by mobility of the metal particles on the support surfaces. Hence, to solve the sintering problem, the encapsulation of the metal nanoparticles within oxide architectures would minimise agglomeration and ensure catalyst recyclability. The validity of this approach was shown, for example, by McFarland and co-workers, comparing the catalytic performance of Pd particles deposited on the outer surface silica (Pd/SiO_2_), or encapsulated within the silica inner porosity (Pd@SiO_2_) [12].

In this context, we have recently introduced a new catalyst series made of Pd and Pt nanocrystals encapsulated in one-step within the sol–gel cages of mesoporous organosilica xerogels. These materials are highly selective mediators in a number of important reactions including carbon-carbon coupling [13], debenzylation [14], highly selective hydrogenation of functionalized nitroarenes [15] and vegetable oils [16], and hydrosilylation of olefins [17]. Applications are not limited to this broad class of reactions and we are continuing to investigate new reactions and synthetic applications.

Work reported in this account investigates the structural origins of the enhanced performance of these new entrapped metal catalysts by Diffuse Reflectance Infrared Fourier Transform (DRIFT) spectroscopy, which is a powerful spectroscopic technique to investigate the molecular structure of these materials, revealing the subtle structural factors affecting their performance. Surface methods, indeed, are not suitable to investigate these materials due to the sol–gel encapsulation of the active species within the pores of the matrix.

## Results and discussion

**Figure 1:**
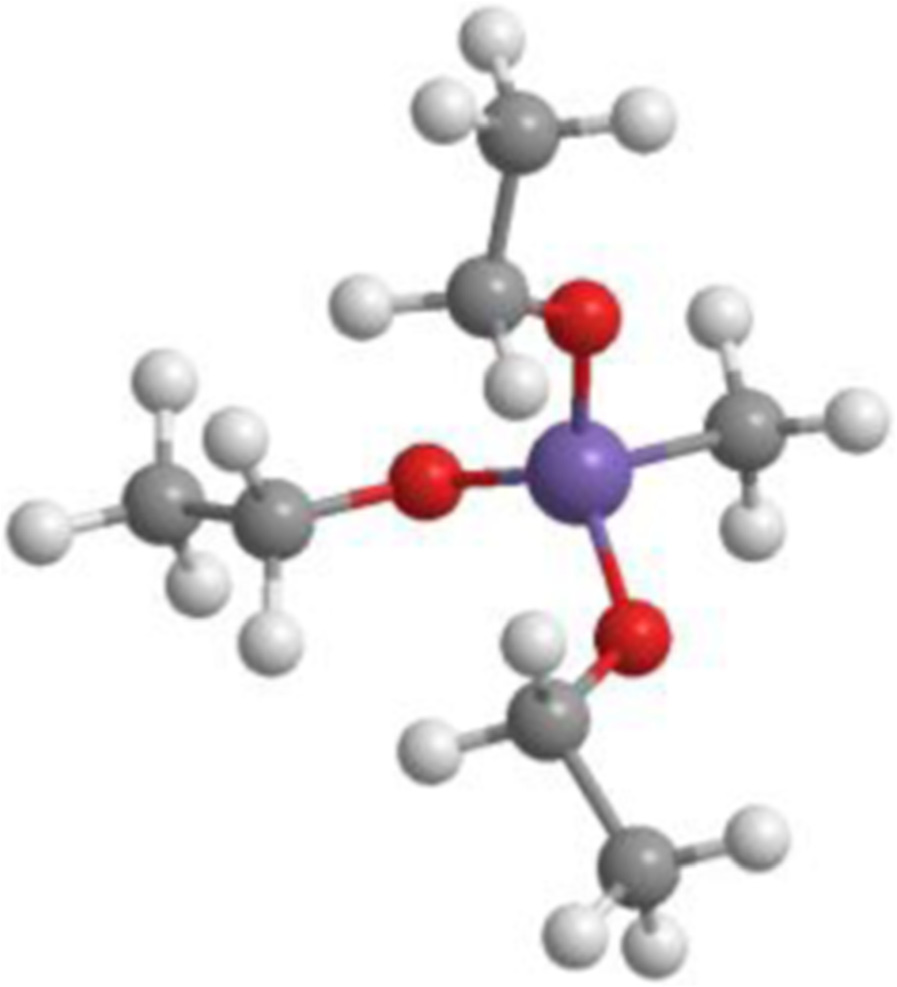
The precursor methyltriethoxysilane (MTEOS).

These novel catalytic materials typically contain Pd(0) or Pt(0) as the nanophase in hybrid alkyl-modified silica materials. For example, two catalysts made from methyl-triethoxysilane (MTEOS, Figure 1) doped with Pd(0) and Pt(0) were prepared according to the hydrolytic polycondensation process for the precursor MTEOS:

-EtOH

$${\mathrm{MeSi}\left(\mathrm{OEt}\right)}_{3}{+\mathrm{catayst}+H}_{2}O\to \mathrm{catayst}@{\left[{\mathrm{Me}‒\mathrm{SiO}}_{n}H_{m}{\left(\mathrm{OEt}\right)}_{q}\right]}_{p}\left(1\right),\mathrm{unbalanced}$$

Efficient entrapment of Pd or Pt nanoparticles is achieved by removing the released alcohol, using rotavapor distillation. Classical sol–gel encapsulation based on hydrolytic polycondensation of Si alkoxides releases large amounts of alcohol that rapidly reduce the Pd^2+^/Pt^2+^ ions to bulk Pd(0)/Pt(0). The latter metal species are catalytically inactive, as only nanostructured MNPs are able to catalyze reactions, such as Pd nanoparticles mediating C-C coupling reactions [8].

**Figure 2:**
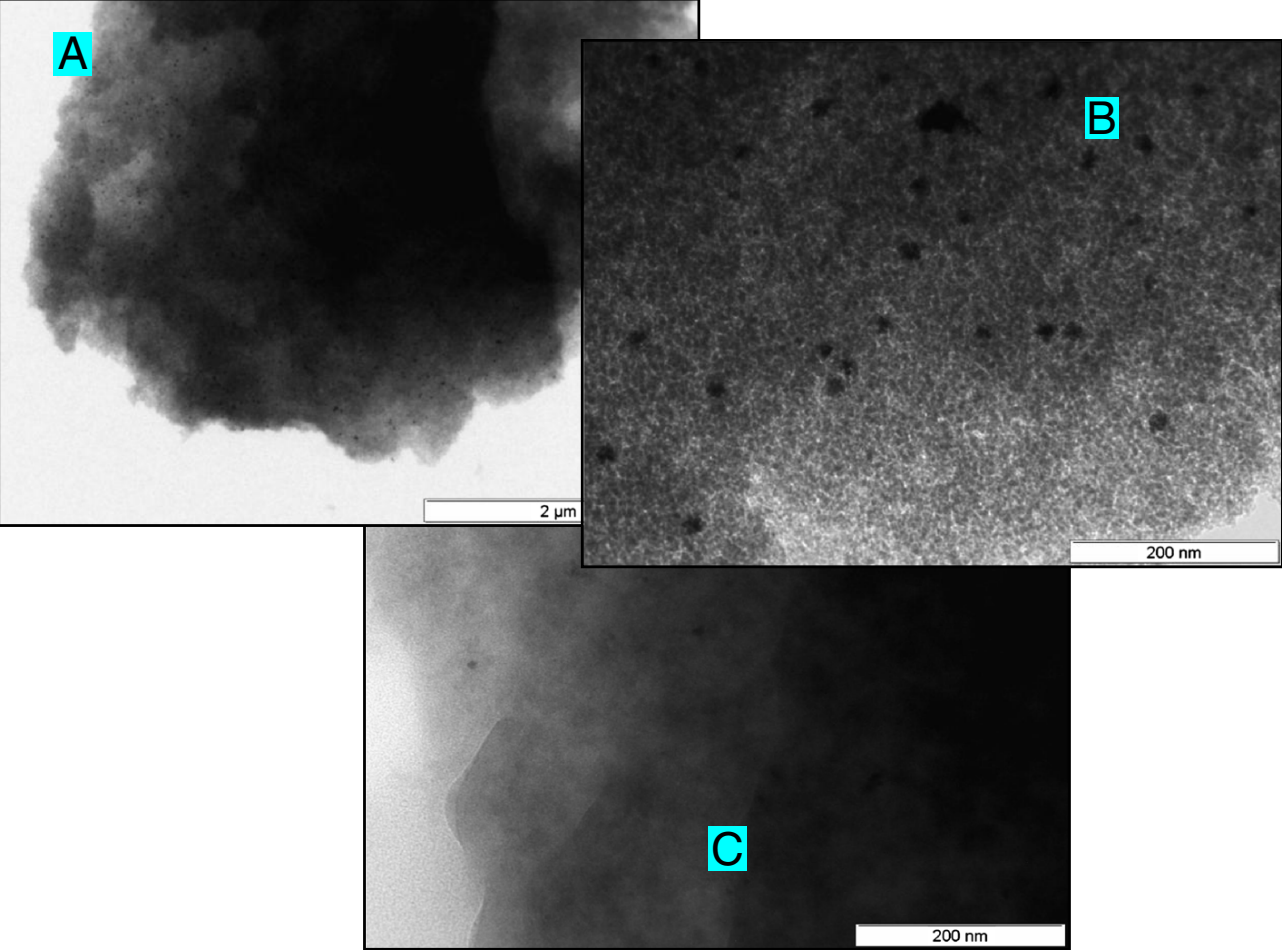
**TEM images of the microstructure of: A and B – Silia** ***Cat *** **Pt**^**0**^**; C – Silia** ***Cat *** **Pd**^**0**^**.**

Here instead, the resulting alcohol-free sol is doped with a Pd(II) or Pt(II) species, and undergoes further basic or acid catalyzed polycondensation to yield a mesoporous hydrogel that is dried under mild conditions to afford a xerogel doped with Pd^2+^/Pt^2+^. The latter material is chemically reduced under mild conditions to yield a mesostructured encapsulated catalyst in which the MNPs are physically and chemically stabilized, affording materials that can safely be used, without the need to exclude air or moisture. The TEM images clearly show the amorphous structure of the ORMOSIL embedding matrix structure in either catalyst. For Silia*Cat* Pt^0^ (Figures 2A and Figure 2B), there is a considerable density of metallic Pt^0^ nanocrystallites with average diameter of ~16 nm, whereas for Silia*Cat* Pd^0^ both the density and dimensions of the Pd^0^ crystallites are smaller (Figure 2C), pointing to a size of ~8 nm.

**Figure 3:**
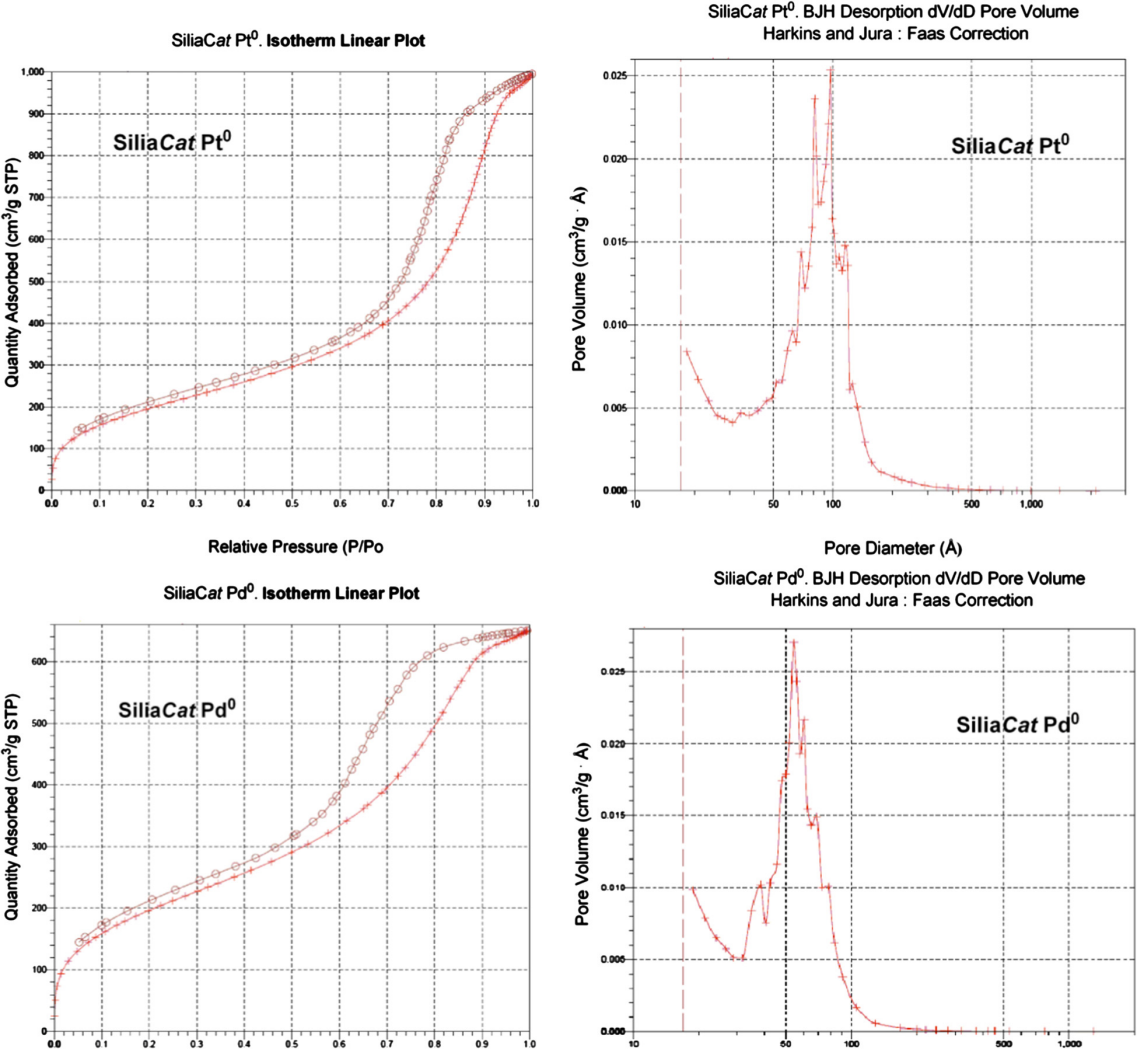
**N**_**2 **_**adsorption-desorption isotherms and mesopore size distributions from BJH analysis of the desorption isotherms of Silia** ***Cat *** **Pt**^**0 **^**(*top*) and Silia** ***Cat *** **Pd**^**0 **^**(*bottom*).**

The N_2_ adsorption-desorption isotherms at 77 K of the two catalysts are shown and compared in Figure 3. For pore size evaluation we used the equation of Harkins and Jura [18]. Hence, the graphs next to the BET reports are the desorption *dV*/*dD* pore volume: Harkins and Jura plot with FAAS correction.

According to the IUPAC classification, the isotherms are type IV with hysteresis loops close to type H1, characteristic of capillary condensation in open cylindrical mesopores between spheroidal particles of fairly uniform array [19]. The mesopore size distribution using the BJH algorithm (that assumes a cylindrical pore shape) has a maximum population at ~9 nm for Silia*Cat* Pt^0^ and 6 nm for Silia*Cat* Pd^0^. The values of parameter *c* in the BET equation (99 for Silia*Cat* Pd^0^ and 79.3 for Silia*Cat* Pt^0^) and the *t*-plot analysis (Harkins and Jura) both indicate that there is no contribution from micropores in either sample. The textural parameters are summarized in Table 1.

**Table 1:** **Textural parameters of Silia** ***Cat *** **Pd**^**0 **^**and Silia** ***Cat *** **Pt**^**0 **^**from the N**_**2 **_**adsorption-desorption isotherms**

**Sample**	**BET specific surface area (m**^**2**^**/g)**	**Total pore volume (cm**^**3**^**/g)**^*a*^	**BJH desorption average mesopore diameter (nm)**^*b*^	**Metal loading (mmol/g)**
Silia*Cat* Pd^0^	706 ± 4.1	1.01	5.7	0.05
Silia*Cat* Pt^0^	712 ± 2.9	1.54	8.7	0.1

^a^The total pore volume is calculated from the maximum amount of N_2_ adsorbed.

^*b*^The average mesopore diameter is calculated from *d* = *4 V*/*S* (*BET surface area*).

Both structures have a very large surface area, exceeding 700 m^2^/g. Silia*Cat* Pd^0^, however, has significantly lower total pore volume. Accordingly, the average mesopore size is smaller for Silia*Cat* Pd^0^, but the size distribution is in either case peaked, pointing to negligible populations of smaller and larger pores.

**Figure 4:**
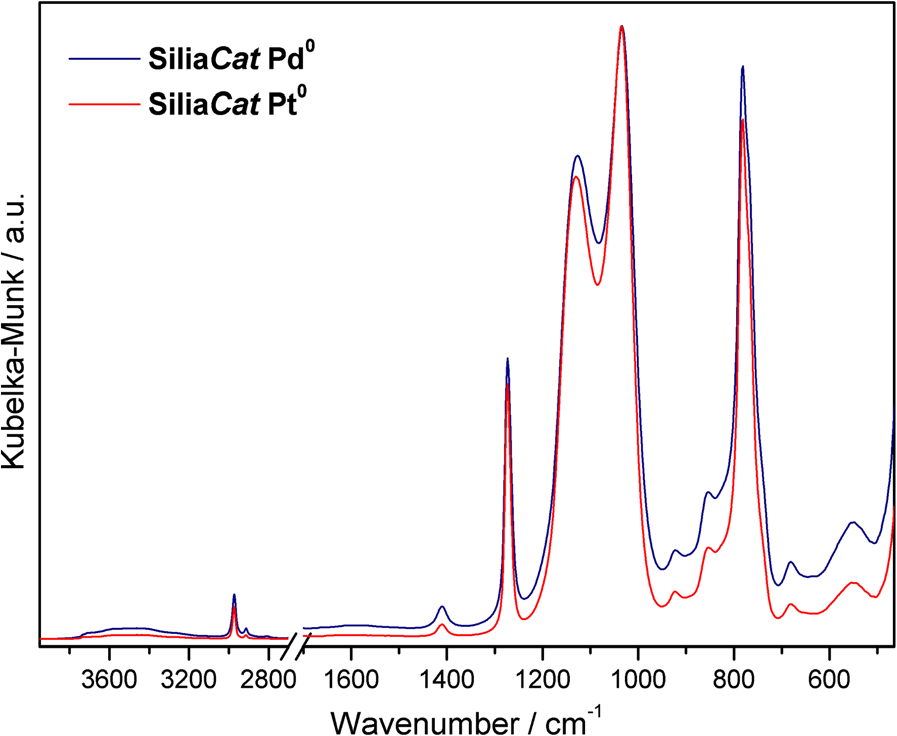
**DRIFT spectra of Silia** ***Cat *** **Pd**^**0 **^**and Silia** ***Cat *** **Pt**^**0 **^**normalized to the maximum.**

The (DRIFT) spectra for both catalysts are shown in Figure 4.

The spectra of the two catalysts are very similar, with only slight changes in the relative intensities of some bands. The proposed band assignments are summarized in Table 2.

**Table 2:** **Assignments of the visible bands in the DRIFT spectra of Silia** ***Cat *** **Pd**^**0 **^**and Silia** ***Cat *** **Pt**^**0**^

**Wavenumber /cm**^**-1**^		**Assignments [**[20]**,**[21]**]**
**Silia** ***Cat *** **Pt**^**0**^	**Silia** ***Cat *** **Pd**^**0**^	
~3500 (broad)	~3500 (broad)	νO-H
2974_w_	2972_w_	ν_as_(Si)CH_3_
2914_vw_	2914_vw_	ν_s_(Si)CH_3_
1410_w_	1410_w_	δ_as_(Si)CH_3_
1273_S_	1273_m_	δ_s_(Si)CH_3_
1130_VS_	1126_VS_	ν_as_Si-O-Si
1036_VS_	1034_VS_	ν_as_Si-O-Si
924_vw_	922_vw_	νSi-O_d_
852_w_	854_w_	ω(Si)CH_3_
781_VS_	781_VS_	ρ(Si)CH_3_
681_w_	681_w_	νSi-C
546_w_	550_w_	(SiO)_4_ rings

Given the high level of methylation of these matrices, it does not come as a surprise that both catalysts are hydrophobic. This is shown by the very weak ν(O-H) band and absence of the δ(HOH) mode, expected at ~1640 cm^-1^ if any water molecules were adsorbed. The ν(O-H) band is therefore assigned to residual silanol (Si-OH) groups. The very low intensities of this band and of the νSi-O(H) or Si-O^-^ (at ~924 cm^-1^) bands show that the condensation reactions were extremely efficient. In addition, the high wavenumber of the ν(O-H) band maximum (~3500 cm^-1^) indicates that the very few silanol groups are not strongly interacting by hydrogen bonds [22].

The two bands at 2974 and 2914 cm^-1^ are assigned to the stretching modes (antisymmetric and symmetric, respectively) of the methyl groups bonded to a silicon atom. The corresponding deformation modes appear at 1410 and 1273 cm^-1^, respectively. The absence of CH_2_ related bands certifies that hydrolysis of methyltriethoxysilane was complete.

**Figure 5:**
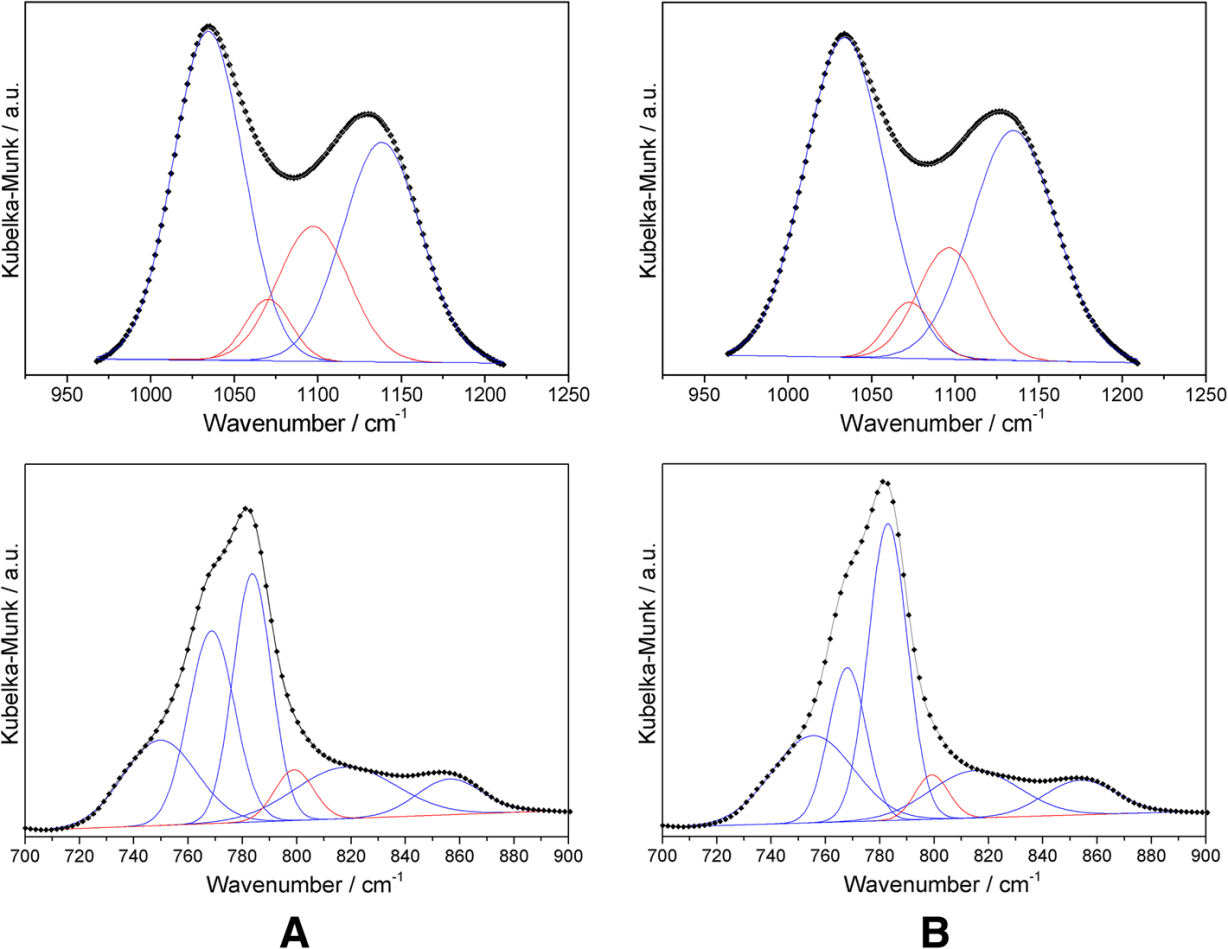
**Deconvolution of the DRIFT spectra in the 950–1250 cm**^**-1 **^**and 700–900 cm**^**-1 **^**regions: A: Silia** ***Cat *** **Pd**^**0**^**; B – Silia** ***Cat *** **Pt**^**0**^**.**

The two catalysts are thus hydrophobic *and* lipophilic. The visible bands in the spectra are assigned in Table 2, but the detailed assignment may only be confidently proposed after band deconvolution in two regions: ^i)^ 950–1250 cm^-1^, which corresponds to the silica structural fingerprint, the ν_as_Si-O-Si mode, split in two observable components, with maxima at ~1130 and ~1036 cm^-1^, cm^-1^; ^ii)^ 700–900 cm^-1^, which apparently corresponds to two bands with maxima at 781 and 852 cm^-1^, but in fact contains a number of overlapping components. The deconvolution of these spectral regions in a sum of Gaussian components was made by a non-linear least squares fitting method, previously described [23]. The deconvolution profiles are shown in Figure 5.

The results concerning the 950–1250 cm^-1^ region are summarized in Table 3, while those regarding the 700 and 900 cm^-1^ region are given in Table 4.

**Table 3:** **Results of the deconvolution of the 950–1250 cm**^**-1 **^**spectral region of the DRIFT spectra: components’ center in cm**^**-1 **^**and relative areas (in%)**

	**Silia** ***Cat *** **Pd**^**0**^		**Silia** ***Cat *** **Pt**^**0**^	
	**(SiO)**_**6 **_**rings**	**(SiO)**_**4 **_**rings**	**(SiO)**_**6 **_**rings**	**(SiO)**_**4 **_**rings**
LO center / cm^-1^	1135	1096	1138	1097
*Area* (^*%*^)	*35*.*8*	*12*.*8*	*32*.*5*	*18*.*3*
TO center / cm^-1^	1034	1073	1035	1070
*Area* (^*%*^)	*46*.*7*	*4*.*7*	*43*.*9*	*5*.*3*
LO-TO splitting/cm^-1^	101	23	103	27
*Relative area%*	*82*.*5*	*17*.*5*	*76*.*4*	*23*.*6*

**Table 4:** **Results of the deconvolution of the 700–900 cm**^**-1 **^**spectral region of the DRIFT spectra: components’ center in cm**^**-1 **^**and relative areas (in%)**

**Assignments**	**Silia** ***Cat *** **Pd**^**0**^		**Silia** ***Cat *** **Pt**^**0**^	
	**Band center / cm**^**-1**^	***Area *** **(%)**	**Band center / cm**^**-1**^	***Area *** **(%)**
ω(Si)CH_3_	856	*6*.*8*	854	*7*.*9*
νSi-C	818	*15*.*3*	815	*12*.*8*
ν_s_Si-O-Si	799	*5*.*8*	799	*4*.*7*
ρ(Si)CH_3_	784	*27*.*7*	783	*35*.*9*
ρ(Si)CH_3_	769	*26*.*2*	768	*17*.*3*
νSi-C	749	*18*.*2*	755	*21*.*4*

**Figure 6:**
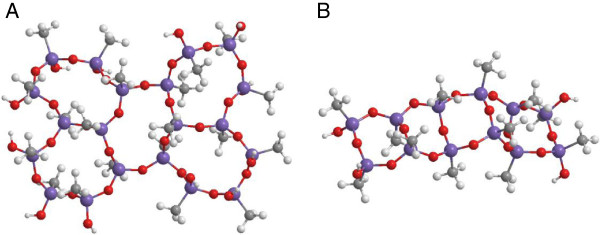
**Schematic representation of a silica cluster containing five cyclic siloxane units: A- six-member [(SiO)**_**6**_**]; B - four member [(SiO)**_**4**_**].**

The ν_as_Si-O-Si mode was decomposed in longitudinal and transverse optical components (LO and TO, respectively) of different siloxane rings: six-member [(SiO)_6_] and four-member [(SiO)_4_]. The relative intensities of the components related to the four-member rings are much smaller indicating that the primary structure of the catalysts is predominantly formed by six-member siloxane rings: both ORMOSIL structures contain ~80% of six-member siloxane rings (Figure 6A) and ~20% of four-member siloxane rings (Figure 6B). These rings indeed are less tensioned, and more suitable to accommodate the extremely high content in methyl groups bonded to the Si atoms. An arrangement of four-member units would yield a much less three dimensional network, as only the terminal Si atoms are able to continue condensation. For undoped ORMOSILs produced from a mixture of trimethoxysilane (TMOS) and methyl-trimethoxysilane (MeTMOS), a similar result was obtained when the methylation degree was higher than 75% [24].

Accordingly, previous preliminary investigation of the Silia*Cat* Pd^0^ structure by solid state NMR [25] has shown that the degree of cross-linking does *not* correlate with the catalytic activity.

Therefore, a predominantly six-member network is perfectly compatible with the porous structure found. Given the low content in four-member siloxane rings, the band at ~550 cm^-1^, which is usually assigned to a coupled mode in these units, must have some contribution from defective structures [26], possibly associated with Pd(II) and Pt(II) species that were not reduced.

Elimination of ethanol from the alcogel mixture contributes to the very large porosity observed in these hydrogel-derived catalysts. Indeed, the capillary tension at the solid-water cage interface is greatly reduced preventing collapse of the gel during drying [27]. Also, elimination of EtOH favours the Si alkoxide monomers hydrolysis and slows down condensation, so that rapid aggregation of the early sol particles is prevented and MTEOS can fully hydrolyse to CH_3_-Si(OH)_3_, which undergoes polycondensation in an open, amorphous structure made predominantly of 6-membered siloxane rings, entrapping Pd or Pt metallic nanophases with the well-known sol–gel stabilization of the nanoparticles.

The spectral region between 700 and 900 cm^-1^ is more difficult to decompose, given the number of overlapping components with close frequencies. The assignment in this region is not straightforward, because Si-C stretching modes are expected, with different frequencies depending on the local structure [28].

An interesting feature of these decompositions is that the ν_s_Si-O-Si band is in fact present, although not resolved. The similarity between the two decompositions confirms the resemblance between the molecular structure of the two catalysts.

The atomic dimension and electronegativity of the metal do not influence the main characteristics of the matrix structure: Pt belongs to the same group and to the following period as Pd (it is much larger and with higher electronegativity). Nevertheless, the ORMOSIL morphology and the dispersion of the metal nanophase are quite different. We emphasize herein the relevance of the support embedding structure in guiding and dictating the access of the reactants to the entrapped nanophase. In other words, encapsulation of the metal nanoparticles within the ORMOSIL structures results in materials that are remarkably more active than traditional catalysts; and this generally allows use of an ultralow amount of valued catalyst under conditions that are milder than those of state-of-the-art processes.

For instance, a 0.05 mol% amount of Silia*Cat* Pd(0) entrapped catalyst can be used to mediate the complete hydrogenation of a wide variety of vegetable oils under hydrogen balloon conditions without *cis*/*trans* isomerisation;^16^ whereas the best Pd catalyst previously known, made of Pd nanoparticles entrapped in the hexagonal porosity of SBA-15 mesoporous silica, mediates less selectively the same conversion at 100°C under 5 atm H_2_[29]. Similar findings have been reported for most of the catalytic processes catalyzed by the Silia*Cat* Pd^0^ and Silia*Cat* Pt^0^ mentioned above.

## Conclusions

The DRIFT investigation of the molecular structure of ORMOSIL-entrapped metal nanoparticles suggests that the mechanism of action of nanoparticles encapsulated in organosilica is similar to that of enzymes. Once the metal nanoparticles are encapsulated and stabilized within the sol–gel cages, it is the hydrophilic-lipophilic balance (HLB) of the matrix that dictates access and optimal catalysis with unprecedented performance, by promoting preferential adsorption of the lipohilic functional group moieties in reacting substrates adsorbed at the surface of the Pd and Pt nanoparticles in reactions as different as hydrogenation of fats, hydrogenation alkenes and nitroarenes, hydrosilylation of olefins and C-C coupling.

The above mentioned reactions concern very large sectors of the chemical industry, many of which continue to use obsolete catalysts such as Ni Raney (hydrogenation of fats), or Pt/C (hydrogenation of olefins). Sol–gel entrapped metal nanophases will give an immense contribute to simplify those processes, whereas new catalysts are being developed capable to target other relevant reactions.

## Experimental section

### Materials synthesis

In a typical preparation, a mixture of methyltriethoxysilane (27 g, 30 mL, 151.4 mmol) and 10 mL of 0.042 M HCl was stirred for 15 minutes. The resulting solution was concentrated with a rotavapor under reduced pressure at 30°C until complete ethanol removal (about 15 minutes). The alcohol-free sol thereby obtained was added with K_2_PdCl_4_ (from 0.004 to 0.02 equivalent) dissolved in H_2_O (from 5 to 10 mL) and 60 mL acetonitrile. This mixture was added with 1 M NaOH (from 0.023 to 0.053 equivalent) to favour gelation that indeed rapidly occurred. The resulting transparent gel was left to dry in air for 4 days after which the xerogel was reduced at room temperature under inert conditions with a solution of sodium triacetoxyborohydride (Pd:Na(AcO)_3_BH = 1:6 molar ratio) in 80 mL THF, washed with THF and H_2_O and dried in air to afford a Silia*Cat* Pd^0^ catalyst. The metal load in each catalyst was measured using the CAMECA SX100 instrument equipped with EPMA analyzer, a fully qualitative and quantitative method of non-destructive elemental analysis of micron-sized volumes at the surface of materials, with sensitivity at ppm level.

### DRIFT analysis

Fourier transform spectroscopy in diffuse reflectance mode was performed in a Mattson RS1 FTIR spectrometer with a Graseby Specac Selector, in the range 400–4000 cm^-1^, at 4 cm^-1^ resolution. The analyses were carried out at ambient temperature and pressure, using a powder catalyst sample as received by the catalysts manufacturer (SiliCycle, Inc.). No further treatment of the catalyst was undertaken prior to measurement.

### TEM analysis

The TEM pictures were obtained in an electron microscope Hitachi H-8100, operated at 200 kV, with a LaB6 filament. The samples were dispersed in ethanol and then dropped onto a Formvar®-coated Cu grid and left to evaporate.

### BET analysis

Nitrogen adsorption and desorption isotherms at 77 K were measured using a an ASAP 2020 system from Micromeritics, analyzing the resulting data with the Tristar 3000 software (version 4,01). The desorption branch was used to calculate the pore size distribution.

## Competing interests

The authors declare that they have no competing interests.

## Authors’ contributions

All authors contributed equally to this work. All authors read and approved the final manuscript.

## References

[B1] Anderson JA Garcia MF Supported Metals in Catalysis 2005 London (UK): Imperial College Press

[B2] Astruc D Lu F Ruiz Aranzaes J Nanoparticles as recyclable catalysts: the frontier between homogeneous and heterogeneous catalysis Angew Chem Int Ed 2005 7 7852 7872 10.1002/anie.200500766 16304662

[B3] Barbaro P Dal Santo V Liguori F Emerging strategies in sustainable fine-chemical synthesis: asymmetric catalysis by metal nanoparticles Dalton Trans 2010 7 8391 8402 10.1039/c002051f 20390200

[B4] Busacca CA Fandrick DR Song JJ Senanayake CH The growing impact of catalysis in the pharmaceutical industry Adv Synth Catal 2011 7 1825 1864 10.1002/adsc.201100488

[B5] Lucarelli C Vaccari A Examples of heterogeneous catalytic processes for fine chemistry Green Chem 2011 7 1941 1949 10.1039/c0gc00760a

[B6] van Gelderen L Rothenberg G Calderone VR Wilson K Shiju NR Efficient alkyne homocoupling catalysed by copper immobilized on functionalized silica Appl Organomet Chem 2013 7 23 27 10.1002/aoc.2933

[B7] Lee I Morales R Albiter MA Zaera F Synthesis of heterogeneous catalysts with well shaped platinum particles to control reaction selectivity Proc Natl Acad Sci U S A 2008 7 15241 15246 10.1073/pnas.0805691105 18832170 PMC2563101

[B8] Reetz MT De Vries JG Ligand-free Heck reactions using low Pd-loading Chem Commun 2004 1559 1563 10.1039/b406719n 15263925

[B9] Jia CJ Schüth F Colloidal metal nanoparticles as a component of designed catalyst Phys Chem Chem Phys 2011 7 2457 2487 10.1039/c0cp02680h 21246127

[B10] Moulijn JA van Diepen AE Kapteijn F Catalyst deactivation: is it predictable? - What to do? Appl Catal A Gen 2001 7 3 16 10.1016/S0926-860X(00)00842-5

[B11] Pagliaro M Pandarus V Ciriminna R Beland F Demma Cara P Heterogeneous versus Homogeneous Palladium Catalysts for Cross-Coupling Reactions ChemCatChem 2012 7 432 445 10.1002/cctc.201100422

[B12] Forman AJ Park JN Tang W Hu YS Stucky GD McFarland EW Silica-Encapsulated Pd Nanoparticles as a Regenerable and Sintering-Resistant Catalyst ChemCatChem 2010 7 1318 1324 10.1002/cctc.201000015

[B13] Pagliaro M Pandarus V Béland F Ciriminna R Palmisano G Demma Carà P A new class of heterogeneous Pd catalysts for synthetic organic chemistry Catal Sci Technol 2011 7 736 739 10.1039/c1cy00119a

[B14] Pandarus V Béland F Ciriminna R Pagliaro M Selective Debenzylation of Benzyl Protected Groups with Silia*Cat* Pd(0) under Mild Conditions ChemCatChem 2011 7 1146 1150 10.1002/cctc.201000420

[B15] Pandarus V Ciriminna R Beland F Pagliaro M A new class of heterogeneous platinum catalysts for the chemoselective hydrogenation of nitroarenes Adv Synth Catal 2011 7 1306 1316 10.1002/adsc.201000945

[B16] Pandarus V Gingras G Béland F Ciriminna R Pagliaro M Selective hydrogenation of vegetable oils over Silia*Cat* Pd(0) Org Process Res Dev 2012 7 1307 1311 10.1021/op300115r

[B17] Ciriminna R Pandarus V Gingras G Béland F Pagliaro M Closing the organosilicon synthetic cycle: efficient heterogeneous hydrosilylation of alkenes over Silia*Cat* Pt(0) ACS Sustainable Chem Engineer 2013 7 249 253 10.1021/sc3001096

[B18] Halsey GD Physical adsorption on non‒uniform surfaces J Chem Phys 1948 7 931 937 10.1063/1.1746689

[B19] Gregg SJ Sing KSW Adsorption, Surface Area, and Porosity 1982 2 New York: Academic Press

[B20] Ciriminna R Ilharco LM Fidalgo A Campestrini S Pagliaro M The structural origins of superior performance in sol–gel catalysts Soft Matter 2005 7 231 237 10.1039/b506021b 32646079

[B21] Lin SY Chang ST Variations of vibrational local modes and electronic states of hydrogenated amorphous silicon carbide under thermal annealing J Phys Chem Solids 1998 7 9 1399 1405 10.1016/S0022-3697(98)00236-4

[B22] Socrates G Infrared and Raman Characteristic Group Frequencies, Tables and Charts 2004 3 Chichester: John Wiley & Sons

[B23] Fidalgo A Ilharco LM Chemical tailoring of porous Silica Xerogels: local structure by vibrational spectroscopy Chem Eur J 2004 7 392 398 10.1002/chem.200305079 14735508

[B24] Fidalgo A Ciriminna R Ilharco LM Pagliaro M Role of the alkyl-alkoxide precursor on the structure and catalytic properties of hybrid sol–gel catalysts Chem Mater 2005 7 6686 6694 10.1021/cm051954x

[B25] Pandarus V Béland F Ciriminna R Demma Carà P Pagliaro M Characterization of Nanostructured Silia*Cat* Pd(0) Catal Letters 2012 7 213 217 10.1007/s10562-011-0741-9

[B26] Kamyia K Yoko T Tanaka K Takeuchi M Thermal evolution of gels derived from CH_3_Si(OC_2_H_5_)_3_ by the sol–gel method J Non-Cryst Solids 1990 7 182 187 10.1016/0022-3093(90)90128-9

[B27] De Witte BM Commers D Uytterhoeven JB Distribution of organic groups in silica gels prepared from organo-alkoxysilanes J Non-Cryst Solids 1996 7 35 41 10.1016/0022-3093(96)00171-8

[B28] King S Bielefeld J Rigidity percolation in plasma enhanced chemical vapor deposited a-SiC:H Thin Films ECS Trans 2010 7 185 194

[B29] Belkacemi K Boulmerka A Arul J Hamoudi S Hydrogenation of vegetable oils with minimum *trans* and saturated fatty acid formation over a new generation of Pd-catalyst Top Catal 2006 7 113 120 10.1007/s11244-006-0012-y

